# Comparison of accuracy of acetabular cup placement using manual versus robotic-assistance in the same patient cohort undergoing robotic-assisted total hip arthroplasty

**DOI:** 10.1007/s11701-026-03497-0

**Published:** 2026-05-28

**Authors:** Adarsh Annapareddy, Tarun Jayakumar, Mujtaba Ansari, Vemaganti Badri Narayana Prasad, A. V. Gurava Reddy

**Affiliations:** Sunshine Bone and Joint Institute, KIMS-Sunshine Hospitals, Hyderabad, India

**Keywords:** Anteversion, Acetabular cup, Inclination, MAKO, Manual, Robotic-assisted, Total hip arthroplasty

## Abstract

Accurate acetabular component orientation is essential for restoring biomechanics and implant longevity in total hip arthroplasty (THA). While robotic assistance has been shown to improve component positioning, comparisons across patient cohorts remain confounded by inter-patient variability. This study aimed to evaluate the accuracy of manual versus robotic acetabular cup impaction using an intra-patient sequential design within the same joint. A prospective observational study was undertaken with 100 robotic-assisted THA (with Mako robotic arm). Following CT-based preoperative planning, a trial acetabular cup was manually impacted and its orientation recorded using intraoperative stereotactic tracking. The trial was then removed, and the definitive cup was implanted using robotic assistance. Deviation from planned patient-specific cup inclination and anteversion was estimated. Overall, the mean age was 44.4 ± 16.1 years, and 71% were males. The mean absolute deviation in anteversion was 4.71 ± 4.210 for manual impaction and 0.93 ± 0.970 for robotic impaction (p < 0.001). The deviation in inclination was 3.5 ± 4.00 with manual and 0.94 ± 1.330 with robotic placement (p < 0.001). Agreement analysis demonstrated that robotic placement had narrower limits and lower bias in cup orientation accuracy compared to manual placement. Robotic-assisted THA achieved significantly greater accuracy and consistency in acetabular cup orientation compared with manual impaction under identical anatomical conditions. While intraoperative precision was improved, further long-term studies are required to determine whether this translates into superior clinical outcomes and implant survivorship.

## Introduction

Total hip arthroplasty (THA) is an effective surgical procedure for restoring mobility and improving the quality of life in patients with advanced hip pathology [1]. Optimal acetabular component orientation is essential for restoring native hip biomechanics and ensuring durable implant performance. Even small angular deviations in inclination or anteversion can influence impingement-free range of motion, liner edge loading, joint stability, and bearing performance [2–4].

The demand for THA is rapidly increasing globally, with India projected to experience one of the highest rates in joint replacement procedures. However, despite this rising volume, there remains a relative paucity of region-specific evidence evaluating advanced technologies, underscoring the need for locally relevant data to guide clinical practice [3]. Additionally, emerging evidence suggests that acetabular orientation may change over time, particularly in young patients, highlighting the importance of achieving accurate initial cup positioning [4].

Conventional acetabular cup placement during THA relies primarily on visual assessment of anatomical landmarks and mechanical alignment guides [3]. This freehand technique is susceptible to several sources of variability, including pelvic motion during impaction, limited intraoperative depth perception, and interference from surrounding soft tissues, and may result in deviation from planned component orientation, with studies reporting up to 70% of acetabular cups positioned outside commonly defined target zones [4].

Technological advances have led to the development of robotic guidance systems aimed at improving the precision of component placement in THA [5]. Robotic-assisted total hip arthroplasty (RA-THA) integrates CT-based preoperative planning with real-time intraoperative feedback and haptic guidance to assist in achieving patient-specific component positioning. By enabling controlled reaming and cup impaction relative to planned orientation, robotic systems have the potential to enhance the accuracy, predictability, and reproducibility of acetabular component placement [6]. Improved accuracy may also provide biomechanical advantages by facilitating optimal load distribution across the articulation, reducing contact stresses, and potentially decreasing polyethylene wear and the risk of implant loosening [7, 8].

Despite these potential benefits, comparisons between manual and robotic acetabular cup placement across different patient cohorts remain influenced by interpatient variability in pelvic tilt, acetabular morphology, and spinopelvic dynamics [2, 9]. To minimize these confounders, the present study employed a unique intra-patient sequential design in which manual and robotic acetabular cup impaction were evaluated within the same joint of each patient undergoing RA-THA. By enabling a direct comparison under identical anatomical and surgical conditions, this approach provides a more robust assessment of the accuracy achievable with robotic assistance. The primary aim of this study was to evaluate the accuracy of robotic acetabular cup placement compared with manual impaction in achieving the planned inclination and anteversion.

## Methods

### Study design

This prospective observational study included consecutive adults who underwent unilateral RA-THA using a CT-based robotic system (MAKO Robotic Arm, Stryker, Kalamazoo, MI) between January 2022 and July 2023 at a high-volume tertiary care center. Patients undergoing conversion arthroplasty were excluded. All procedures were performed by a single senior arthroplasty surgeon to ensure procedural consistency. The study protocol was approved by the institutional ethics committee (SIEC/2022/505) and conducted in accordance with the Declaration of Helsinki [10]. Written informed consent was obtained from all participants.

### Preoperative planning

All patients underwent preoperative CT-based three-dimensional planning using MAKO Hip Software (Version 4.0, Stryker, Mahwah, NJ) to determine optimal acetabular component positioning based on patient anatomy, virtual range of motion, and spinopelvic parameters. The mean preoperative planned acetabular anteversion was 21.19^0^ ± 3.74^0^, and the mean planned inclination was 40.12^0^ ± 0.92^0^.

### Surgical technique

All procedures were performed using the standard posterior approach with the patient in the lateral decubitus position [11]. Following pelvic and femoral registration, the acetabular preparation was performed under robotic guidance. After reaming, an uncemented press-fit trial cup was first impacted manually. During manual impaction, no mechanical alignment guides or intraoperative fluoroscopy were used. The cup inclination and anteversion were recorded intraoperatively using stereotactic tracking arrays by probing five equidistant points along the rim of the cup. To ensure measurement consistency, all 5 points were obtained along the same concentric circle at the inner border of the outer flanges of the trial cup.

**Fig. 1:**
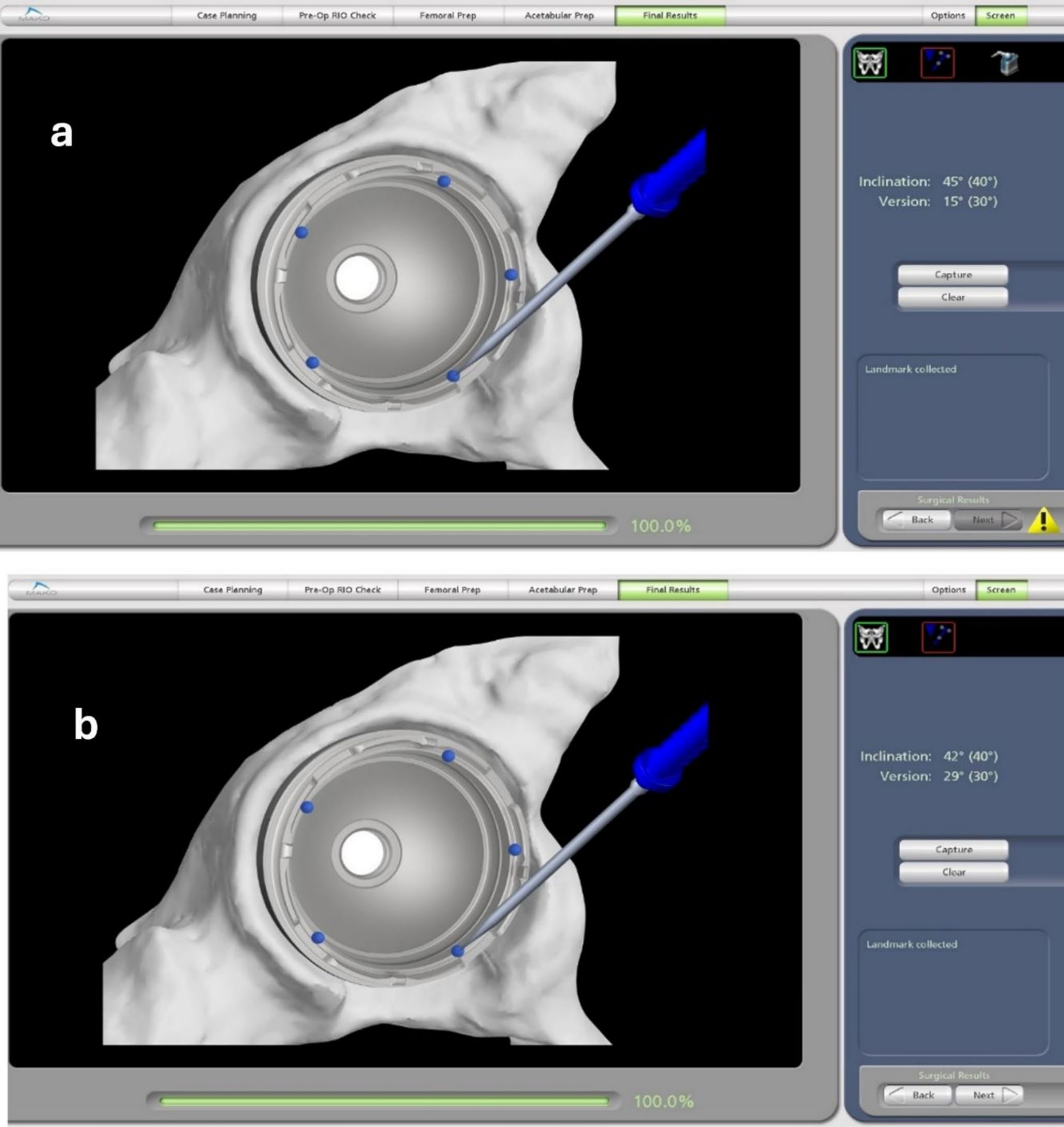
Intraoperative acquisition of reference points on the (**a**) trial cup and (**b**) definitive acetabular cup during RA-THA

**Fig. 2:**
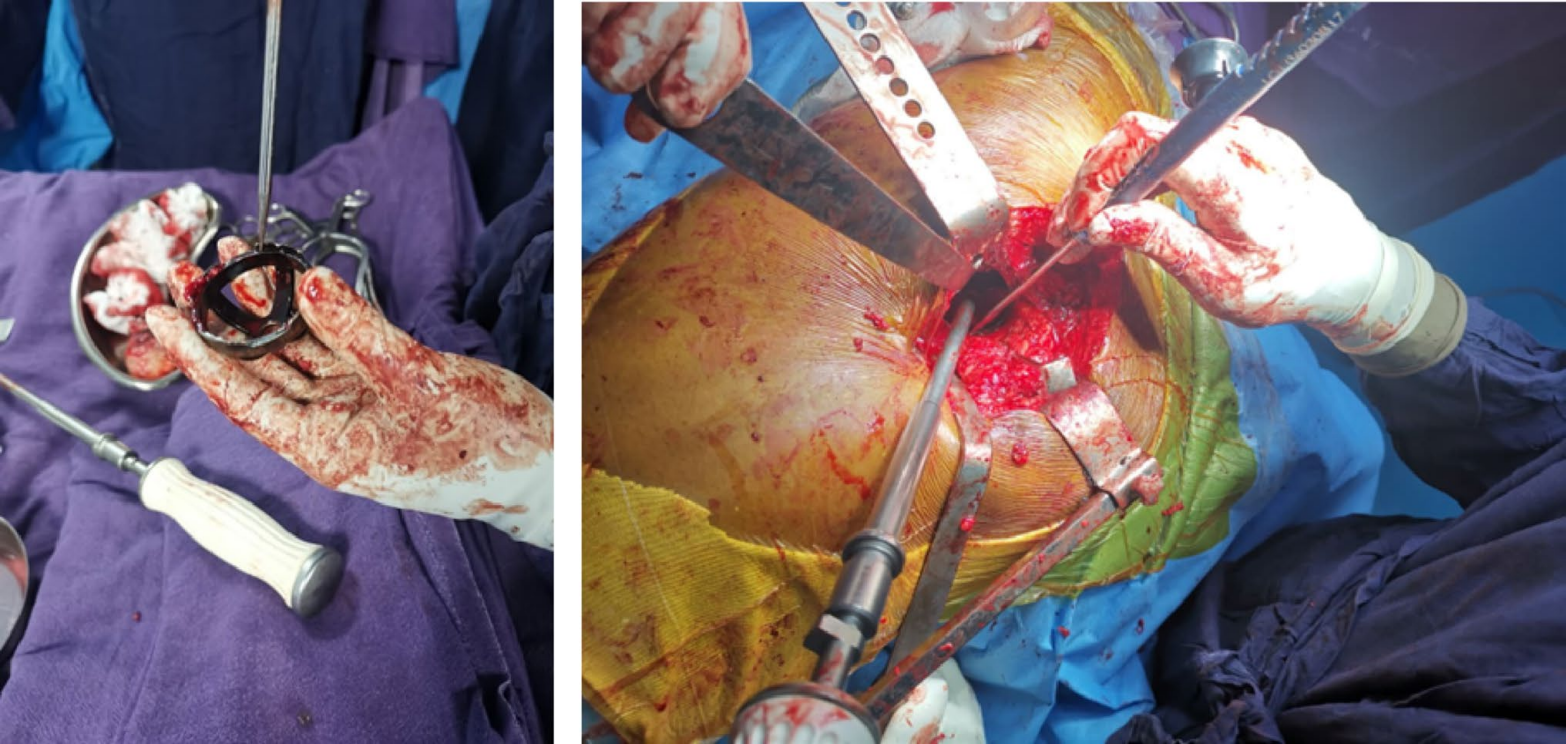
(Left) Image showing points along the cup where 5 points are recorded on the same concentric circle, between the inner border of the outer flanges of the trial cup. (Right) Intra-operative image showing the cup position recording after manual cup impaction

The trial component was subsequently removed, and the definitive acetabular component was implanted using robotic-assisted impaction, with haptic guidance to constrain positioning within the predefined target orientation. Orientation of the final component was recorded using the same measurement protocol to ensure consistency (Figs. 1 and 2). All patients received uncemented Stryker Accolade II femoral and Trident acetabular components.

### Statistical analysis

Continuous variables were summarized as mean ± standard deviation or median (range), as appropriate. Paired comparisons between manual and robotic measurements were evaluated using the paired t-test. Categorical variables were reported as frequencies and percentages. Binary paired data were analyzed using McNemar’s test. Agreement between planned and achieved cup orientations was assessed using Bland–Altman analysis to calculate mean bias and 95% limits of agreement. As no previously published studies have employed a similar intra-patient comparative design, the sample size was determined pragmatically, based on previous RA-THA accuracy studies. Statistical significance was defined as a two-sided *p* < 0.05. All analyses were performed using R software (version 4.4.1; R Foundation for Statistical Computing, Vienna, Austria).

## Results

A total of 100 patients underwent RA-THA with a mean age of 44.4 ± 16.1 years and 71% males. The mean body mass index (BMI) was 24.7 ± 2.6 kg/m^2^. The right hip was operated in 55 patients and the left in 45 patients. The predominant indications for surgery were avascular necrosis of the femoral head and fractured neck of the femur (Table 1).

**Table 1:** Demographic and clinical characteristics of patients (*N* = 100)

Variable	Value
Age (y), Mean ± SD	44.4 ± 16.1
Body Mass Index (kg/m²), Mean ± SD	24.7 ± 2.6
Male; N (%)	71 (71)
Female; N (%)	29 (29)
THA Laterality	
Right; N (%)	55 (55)
Left; N (%)	45 (45)
Diagnosis	
Avascular Necrosis; N (%)	64 (64)
Fracture Neck of Femur; N (%)	18 (18)
Primary Osteoarthritis; N (%)	7 (7)
Failed Intertrochanteric Fracture Fixation; N (%)	11 (11)

### Cup position accuracy

**Fig. 3:**
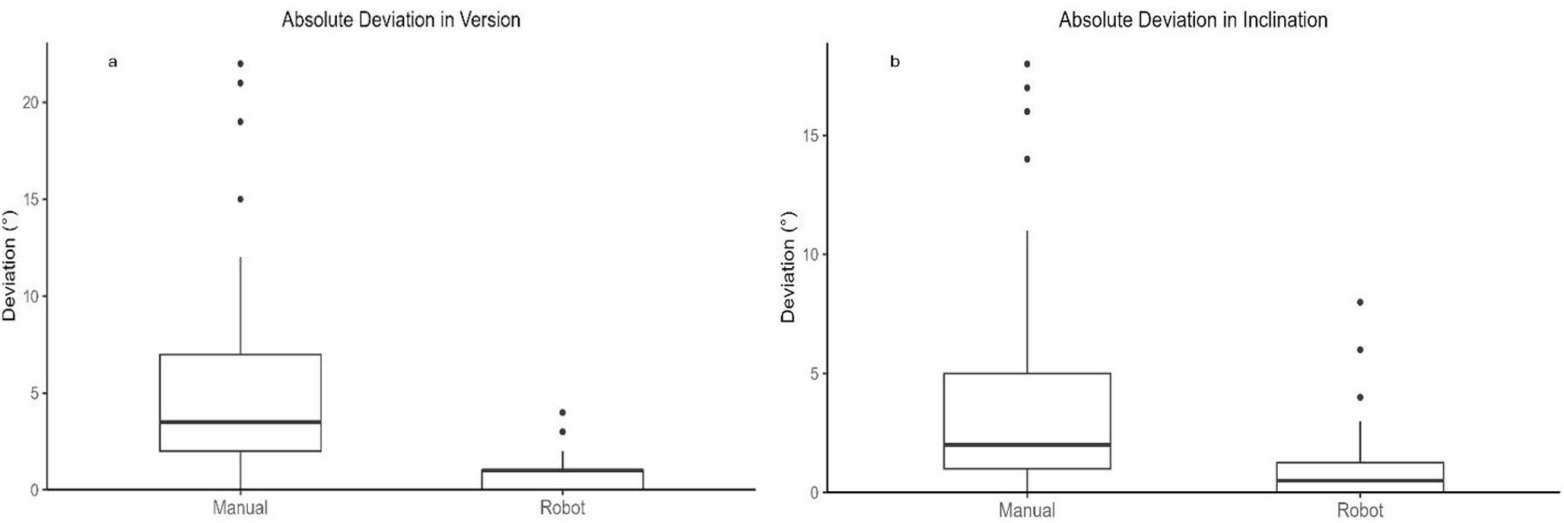
Box-and-Whisker plot showing absolute deviation from plan in version (**a**) and inclination (**b**) using manual and robotic approaches

The absolute deviation in anteversion was 4.71 ± 4.21^0^ for manual placement and 0.93 ± 0.97^0^ for robotic placement (*p* < 0.001). The absolute deviation in inclination was 3.5 ± 4.0^0^ with manual placement and 0.94 ± 1.33^0^ with robotic placement (*p* < 0.001; Fig. 3). The proportion of cups placed within ± 2^0^ of the planned anteversion was 39% for manual and 95% for robotic placement (*p* < 0.001), while within ± 5^0^ it was 66% and 100% (*p* < 0.001), respectively. Similarly, for inclination, 54% of cups placed manually and 90% placed robotically were within ± 2^0^ of the planned values (*p* < 0.001), whereas within ± 5^0^, the corresponding proportions were 80% and 98% (*p* < 0.001), respectively, as shown in Table 2.

**Table 2:** Accuracy of manual and robotic cup placement

Parameter	Manual	Robotic	*p*-value
Absolute deviation in version	4.71^0^±4.21^0^	0.93^0^±0.97^0^	< 0.001*
Absolute deviation in inclination	3.5^0^±4.0^0^	0.94^0^±1.33^0^	< 0.001*
Version Within 2^0^	39%	95%	< 0.001^#^
Version Within 5^0^	66%	100%	< 0.001^#^
Inclination Within 2^0^	54%	90%	< 0.001^#^
Inclination Within 5^0^	80%	98%	< 0.001^#^

*paired t-test,* #McNemar test*

### Agreement between planned and measured cup placement

**Fig. 4:**
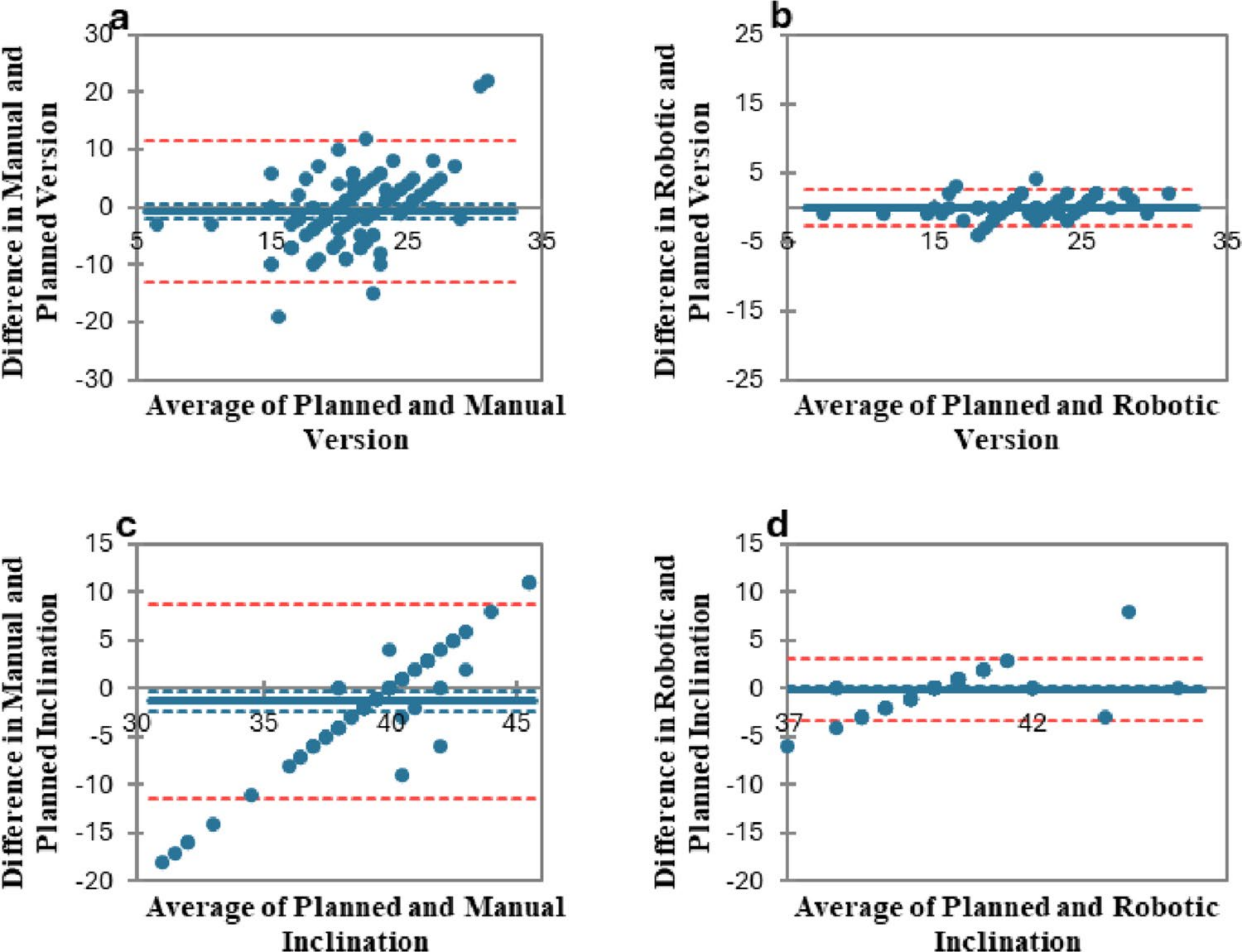
Bland-Altman plot on agreement between the pre-operative plan and the measured version and inclination of the acetabular cup with the 95% confidence intervals (CI): **a**-version-manual; **b**-version-robotic; **c**-inclination-manual; **d**-inclination-robotic

Bland-Altman plots (Fig. 4) showed a wider spread and greater bias for manual inclination and version. In contrast, robotic placement demonstrated minimal bias and narrower limits of agreement for both inclination and version, reflecting higher accuracy according to the preoperative plan.

## Discussion

The present study demonstrated that robotic-assisted total hip arthroplasty achieved more accurate and consistent acetabular cup orientation compared with manual impaction. By evaluating robotic and manual impaction techniques sequentially within the same joint of each patient, this study uniquely assessed the effect of robotic guidance under identical anatomical and surgical conditions. To the best of our knowledge, this is the first investigation comparing the accuracy of robotic-assistance and manual impaction within the same hip, rather than across separate patient cohorts, thereby allowing a more direct evaluation of the technological contribution to component positioning.

Accurate acetabular cup orientation remains a key determinant of biomechanical restoration, and long-term implant performance in THA [1]. Consistent with this, prior evidence shows that patients with dislocations are less likely to have components positioned within the Lewinnek safe zone. In primary THA, only 24% of dislocating hips were positioned within the safe zone compared to 50% of non-dislocating controls (*p* < 0.001), with dislocators exhibiting higher inclination (47.5^0^ vs. 45.1^0^, *p* = 0.01) and lower anteversion (17.6^0^ vs. 20.5^0^, *p* = 0.04) [12].

Despite the widespread adoption of target ‘safe zones’, achieving consistent cup orientation with conventional freehand techniques remains challenging [13, 14], as cup placement relies largely on visual assessment of anatomical landmarks and mechanical alignment guides, making it inherently susceptible to intraoperative variability [3]. Such limitations underscore the importance of technologies that can improve the precision and reproducibility of component alignment.

With the rapidly increasing volume of joint arthroplasty procedures in India, the importance of generating robust, region-specific evidence to support emerging technologies such as robotic-assisted surgery is becoming increasingly evident [3]. In this context, the present study contributes relevant data on the accuracy of robot-assisted acetabular orientation. Furthermore, emerging long-term data indicate that acetabular orientation may not remain static, particularly in younger patients, who can experience changes in pelvic tilt over time, resulting in clinically meaningful alterations in cup inclination and anteversion [4]. Notably, more than 60% of the patients in the present study were younger individuals. The improved accuracy and consistency observed with RA-THA may therefore have important implications for maintaining optimal functional orientation over time in this patient population.

Robotic-assisted techniques address several of these limitations by integrating preoperative CT-based planning with real-time intraoperative feedback and haptic guidance [13]. During implantation, the robotic platform provides continuous positional tracking and constrains instrument movement within predefined boundaries, enabling surgeons to detect and correct alignment deviations before final component impaction [15, 16]. This is reflected in the present study, which demonstrates improved predictability and a reduction in outlier positioning compared with manual techniques.

This real-time feedback mechanism not only enhances accuracy but also improves procedural consistency and surgeon confidence. In addition, robotic guidance may be particularly advantageous in technically demanding scenarios such as dysplastic acetabula, severe deformity, obesity, and minimally invasive surgical approaches, where visualization and anatomical orientation can be more challenging [17–19]. Compared with other positioning technologies, including computer-assisted navigation or fluoroscopic guidance, robotic systems provide a more integrated workflow and tactile control during reaming and cup impaction, facilitating reproducible execution of the preoperative plan and reducing the chances of outlier component placement [15, 20, 21].

Our findings are consistent with previous reports demonstrating improved accuracy and reproducibility of acetabular component placement related to both conventional safe zones and individualized functional alignment targets [2, 11, 22]. Sicat et al., in a prospective multicenter study involving 251 primary THA patients, demonstrated that functional safe zone planning was executed more accurately with robotic-assistance compared to both computer-assisted navigation and manual instrumentation [22]. Similarly, a prospective cohort study comparing robotic-assisted and conventional THA reported superior restoration of the native center of rotation and more consistent acetabular component placement within Lewinnek’s safe zone when robotic guidance was used [2]. Previous studies have further shown that more than 90% of robotic-assisted cases fall within 5° of the preoperative target, with predictive intervals for inclination and version as narrow as ± 3.5° and ± 3.6°, respectively, reflecting strong agreement between planned and achieved orientations [1, 4, 11, 19, 23]. Together, these data corroborate the precision and reproducibility achieved with robotic assistance, as demonstrated in the current study using the Mako system.

However, some studies have reported discrepancies between planned and achieved anteversion despite robotic assistance [24]. These differences may largely reflect methodological variation, as such analyses compare preoperative planning with postoperative CT measurements across different patient cohorts. In contrast, the present study employed an intra-patient sequential comparison in which manual and robotic impaction were assessed within the same joint, minimising confounding factors. Furthermore, postoperative CT-based measurements may be influenced by functional pelvic tilt and patient positioning during imaging, whereas the intraoperative stereotactic measurements used in the present study more directly reflect implant orientation at the time of placement.

Beyond radiographic precision, emerging clinical evidence suggests that improved component positioning with robotic assistance may translate into enhanced early functional outcomes and reduced blood loss due to the single acetabular ream feature of CT-based robotics [25]. Studies evaluating CT-based robotic-assisted implantation of Trident II acetabular components using the MAKO robotic platform have demonstrated sustained improvements with a mean postoperative abduction angle of 42.9 ± 3.4^0^ and minimal changes at 6 months (1.8 ± 1.7^0^), accompanied by minimal variation in horizontal (1.4 ± 1.2 mm) and vertical (1.0 ± 0.9 mm) offsets, indicating minimal component migration. These findings, together with sustained improvements in patient-reported outcome measures, including the Western Ontario and McMaster Universities Arthritis Index, PROMIS-10 physical score, and Hip Disability and Osteoarthritis Outcome Score-Joint replacement scores, from 6 months through 2 years, support the clinical relevance of precise robotic-assisted cup positioning [26]. Consistent with these observations, a matched cohort study with a minimum two-year follow-up demonstrated a significantly greater proportion of acetabular cups positioned within established safe zones using RA-THA compared with manual techniques, including the Lewinnek safe zone (98.8% vs. 78.8%, respectively; *p* < 0.001) and the Callanan safe zone (92.9% vs. 60%, respectively; *p* < 0.001). Correspondingly, clinical outcomes were superior in the robotic-assisted group, with significantly higher Harris Hip Scores (*p* < 0.001) and Forgotten Joint Scores (*p* = 0.003) [27].

Long-term evidence further substantiates the potential clinical relevance of accurate robotic-assisted component positioning. A 10-year analysis of robotic-assisted primary THA demonstrated high accuracy of acetabular cup placement, with more than 90% of components positioned within established safe zones, no reported dislocations, and a survivorship free from revision exceeding 97% [28]. Meta-analytic evidence has also demonstrated improved patient-reported outcomes including Forgotten Joint Scores and Oxford Hip Scores, following Mako robotic-assisted THA compared with conventional THA, without an increased risk of revisions or complications [23]. Collectively, these findings suggest that accurate acetabular cup placement achieved with robotic assistance confers clinical relevance, contributing to sustained improvements in clinical outcomes associated with pain, functional recovery, and joint awareness following THA.

The present study has certain limitations. First, the sequential comparison design may introduce order bias because manual impaction was always performed before robotic placement. Second, all procedures were performed by a single experienced surgeon, which ensured procedural consistency but did not allow assessment of inter-surgeon variability. Third, although improved intraoperative accuracy was demonstrated with robotic assistance, the study did not evaluate long-term clinical outcomes. Further investigations should evaluate these findings across larger multicenter cohorts to confirm their generalizability. Studies assessing multi-surgeon reproducibility and integrating radiographic accuracy with patient-reported outcome measures and implant survivorship will be particularly important to further clarify the clinical impact of RA-THA.

## Conclusion

Robotic-assisted total hip arthroplasty enabled a patient-specific approach to acetabular cup placement, achieving significantly greater accuracy and consistency in inclination and anteversion compared with the manual technique under identical anatomical and surgical conditions. Robotic guidance substantially reduced deviation from the planned component orientation, improving the precision and reproducibility of acetabular cup positioning. Further long-term studies are required to determine whether these technical advantages translate into improved clinical outcomes and prosthesis survivorship.

## Data Availability

No datasets were generated or analysed during the current study.
